# Timcodar (VX-853) Is a Non-FKBP12 Binding Macrolide Derivative That Inhibits PPAR*γ* and Suppresses Adipogenesis

**DOI:** 10.1155/2016/6218637

**Published:** 2016-04-14

**Authors:** Terry D. Hinds, Kezia John, Lucien McBeth, Christopher J. Trabbic, Edwin R. Sanchez

**Affiliations:** ^1^Center for Hypertension and Personalized Medicine, Department of Physiology & Pharmacology, University of Toledo College of Medicine, Toledo, OH 43614, USA; ^2^Center for Drug Design and Development, Department of Medicinal & Biological Chemistry, University of Toledo College of Pharmacy and Pharmaceutical Sciences, Toledo, OH 43606, USA; ^3^Center for Diabetes and Endocrine Research, Department of Physiology & Pharmacology, University of Toledo College of Medicine, Toledo, OH 43614, USA

## Abstract

Nutrient overload and genetic factors have led to a worldwide epidemic of obesity that is the underlying cause of diabetes, atherosclerosis, and cardiovascular disease. In this study, we used macrolide drugs such as FK506, rapamycin, and macrolide derived, timcodar (VX-853), to determine their effects on lipid accumulation during adipogenesis. Rapamycin and FK506 bind to FK506-binding proteins (FKBPs), such as FKBP12, which causes suppression of the immune system and inhibition of mTOR. Rapamycin has been previously reported to inhibit the adipogenic process and lipid accumulation. However, rapamycin treatment in rodents caused immune suppression and glucose resistance, even though the mice lost weight. Here we show that timcodar (1 *μ*M), a non-FKBP12-binding drug, significantly (*p* < 0.001) inhibited lipid accumulation during adipogenesis. A comparison of the same concentration of timcodar (1 *μ*M) and rapamycin (1 *μ*M) showed that both are inhibitors of lipid accumulation during adipogenesis. Importantly, timcodar potently (*p* < 0.01) suppressed transcriptional regulators of adipogenesis, PPAR*γ* and C/EBP*α*, resulting in the inhibition of genes involved in lipid accumulation. These studies set the stage for timcodar as a possible antiobesity therapy, which is rapidly emerging as a pandemic.

## 1. Introduction

**Figure 1:**
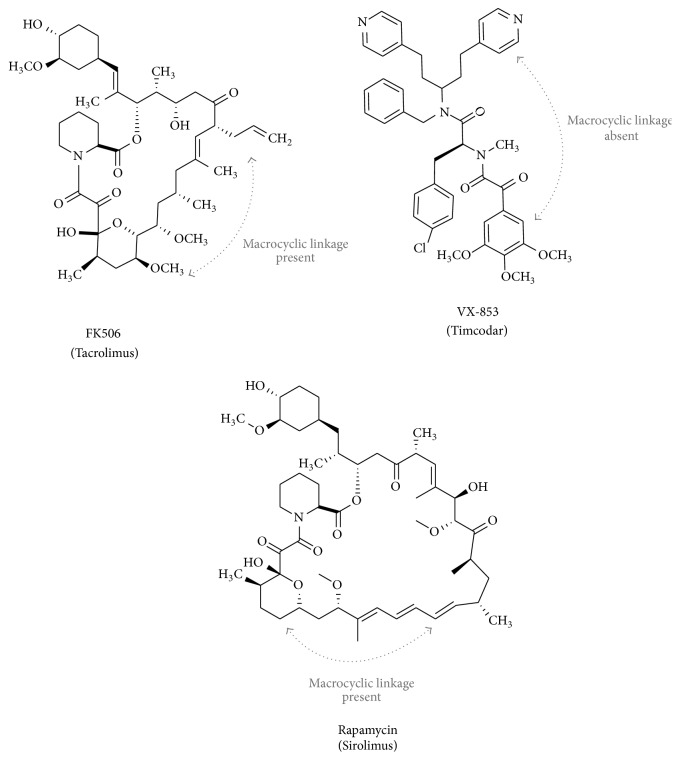
Structural comparison of FKBP binding macrolides and timcodar. Chemical structures of FK506 (Tacrolimus), timcodar (VX-853), and rapamycin (Sirolimus). Note that VX-853 is not a macrocycle (highlighted by the presence and absence of a “macrocyclic linkage”).

Drugs that are inhibitors of the adipogenic process are of much interest due to their capacity to reduce lipid accumulation in obese patients. Due to genetic factors and diets high in fat, obesity is on the rise and quickly emerging as an epidemic. The total excess cost related to obesity is estimated to be $254 billion/year in US health expenditures by 2030. We have recently shown that tetratricopeptide repeat (TPR) proteins, such as protein phosphatase 5 (PP5) [1] and FK506 binding proteins (FKBPs) 51 [2, 3] and 52 [4] (FKBPs range in molecular weight from 12 to 135 kDa [5]), are important regulators of lipid metabolism. Macrolide analogues, FK506 and rapamycin (Figure 1), are immunosuppressive drugs that target FKBP proteins. They have been shown to be effective in suppression of the immune system by the inhibition of the small molecular weight FKBP, FKBP12 [6]. These drugs have been used for suppression of the immune system during transplantation to aid in organ acceptance. However, their potential roles in the treatment of obesity are only now becoming known. In the past decade, low-grade inflammation has been observed as a possible cause of obesity and type II insulin-resistant diabetes [7]. Thus, drugs that target the FKBPs may serve as potential therapies that ameliorate excess lipid accumulation and low-grade inflammation.

Unlike the smaller FKBPs, FKBP51 and FKBP52 contain three TPR domains within their structure, which allows them to bind to the chaperone heat-shock protein 90 (HSP90) and nuclear receptor complexes [8, 9]. We have shown that reduced FKBP52 expression in mice challenged with a high-fat diet resulted in exacerbated diet-induced lipid accumulation in the liver (hepatic steatosis) as well as insulin and glucose intolerance [4]. FKBP51, on the other hand, had an inverse function and the loss resulted in reduced lipid accumulation in cellular models of adipogenesis [2, 3]. Utilizing the 3T3-L1 murine adipocyte model, Yeh et al. showed that rapamycin, and not FK506, was a potent inhibitor of adipogenesis [10]. The mechanism that these macrolides use to reduce lipid accumulation remains unknown. FK506 bound FKBP12 inhibits calcineurin, whereas the rapamycin-FKBP12 interaction had no effect [11–13]. Calcineurin acts as a calcium-dependent molecular switch that negatively regulates adipocyte differentiation [14]. Later studies show that rapamycin, through FKBP12, inhibits the mammalian target of rapamycin (mTOR), leading to the inhibition of protein synthesis and growth [15, 16]. For these reasons, rapamycin and FK506 have been mostly considered FKBP12 ligands. Rapamycin causes suppression of adipocytic transcription factors, peroxisome proliferator-activated receptor *γ* (PPAR*γ*) and CCAAT/enhancer-binding protein *α* (C/EBP*α*) [17], which regulate fatty acid uptake and* de novo* lipid synthesis [18].

We have shown that TPR proteins, FKBP51 and PP5, can bind specifically to the PPAR*γ* heteromeric complex and positively regulate receptor activity [1–3]. Upon ligand binding of PPAR*γ*, PP5 enters the nuclear receptor complex to dephosphorylate serine 112 of PPAR*γ*, which is the amino acid in PPAR*γ* shown to control the adipogenic pathway [1, 19]. FKBP51 is bound in the PPAR*γ* complex, but this was only investigated in the ligand-free state [2]. Interestingly, Davies et al. demonstrated that the glucocorticoid receptor (GR) in the native state has a higher affinity for FKBP51, and exchange for FKBP52 takes place when interaction with glucocorticoids occurs [20]. Later studies showed that FKBP52 was a positive regulator of GR and essential for gene regulated activity [9]. The effect of FKBP52 on PPAR*γ* activity remains unknown. However, FK506 and rapamycin have been shown to potentiate the dexamethasone-induced GR response, suggesting that they target not only FKBP12 but also the larger FKBPs [21]. Rapamycin has been shown to bind to the larger FKBP, FKBP51; and mTOR inhibition is determined by the relative expression of the FKBPs [22]. FK506 has been demonstrated to bind both FKBP51 and FKBP52 [23, 24].

The immunophilin macrolide FK506 exerts its potent immunosuppressive effects principally by targeting FKBP12 [6]. With the discovery that FK506 also had neurotrophic activity [25], a need for analogues that are non-FKBP12 ligands has developed. Through the work of Bruce Gold and others, several FK506 analogues devoid of FKBP12 binding capacity have been identified that can fundamentally increase neurite elongation and accelerate nerve regeneration [26]. These properties have been exploited to show that non-FKBP12-binding analogues can be protective against diseases of the nervous system, such as autoimmune encephalomyelitis [27]. Although the neuroprotective mechanism of action for the non-FKBP12-binding compounds is still far from clear, these effects have been attributed to FKBP52, not FKBP12, which leads to disruption of FKBP52-containing nuclear receptor complexes and activation of the extracellular signal-regulated kinase (ERK) pathway [28, 29]. Of particular interest to this work is the compound timcodar (VX-853), a nonimmunosuppressant FK506 derivative developed by Vertex that cannot bind FKBP12 but which is purported to promote neurite outgrowth [30] and improve nerve function in a rat model of drug-induced diabetic neuropathy [31]. A more recent small, clinical trial showed no effect of timcodar on nerve regeneration in patients subjected to standardized nerve injury [32]. However, only healthy patients were used in this trial, leaving open the possibility that timcodar and related drugs may indeed be of benefit under diabetic conditions. Because of timcodar's structural similarity to FK506 derivatives shown to bind FKBP52, we tested its ability to target FKBP52 and FKBP51 and affect the actions of those chaperones on glucocorticoid receptor activity. Through the use of FKBP51 and FKBP52 knockout mouse cell lines, we showed that timcodar rescued the reduced GR activity typically seen in FKBP52 knockout cells, but only when FKBP51 was present, suggesting that FKBP51 may be a direct target of timcodar actions [33]. However, direct biochemical assays using purified fragments of human FKBP51 and FKBP52 have failed to demonstrate timcodar binding to either FKBP [34]. It should be noted that this work used only the FK1 domain containing the peptidyl-prolyl cis-trans isomerase (PPIase) function of the proteins. Because both FKBP51 and FKBP52 contain an additional and closely juxtaposed PPIase-like domain (FK2), it is possible that timcodar may control the FKBPs via the FK2 domain.

In these studies, we show that timcodar inhibited lipid accumulation in 3T3-L1 cells similar to rapamycin and that FK506 had no effect. Interestingly, timcodar robustly suppressed the expression of the master adipogenic regulator, PPAR*γ*, much stronger than rapamycin. These preliminary studies suggest that timcodar may serve as a therapeutic for obesity.

## 2. Materials and Methods

### 2.1. Materials and Cell Lines

The mouse 3T3-L1 preadipocyte cells were routinely cultured and maintained in Dulbecco's Modified Eagle's Medium (DMEM) containing 10% bovine calf serum or FBS with 1% penicillin-streptomycin. FK506 and rapamycin were from Cell Signaling Technology, Inc. (Boston, MA). VX-853 was a gift from Dr. Bruce Gold (Oregon Health and Science University).

### 2.2. Proliferation Assays

3T3-L1 cells (2.5 × 10^4^ cells per well) were plated in 12-well plates in DMEM containing 10% calf serum. The effect of the drugs on growth rate was determined at 48 hours with treatment with 0.1 and 1.0 *μ*M of FK506, rapamycin, or timcodar. Cell proliferation was determined by a calorimetric assay using MTT (3-(4,5-dimethylthiazol-2-yl)-2,5-diphenyltetrazolium bromide) as previously described [35].

### 2.3. Adipogenesis Assay

Adipogenic differentiation of 3T3-L1 cells was achieved by treatment with 1 *μ*M Dex, 830 nM insulin, and 100 *μ*M isobutylmethylxanthine in 10% FBS on Day 0 of the differentiation protocol [1–3]. Upon differentiation, cells were stained with Nile Red to visualize lipid content, and densitometry was used as a direct measure as described in [1–3]. To determine the effect of timcodar on adipogenesis we treated with 0 *μ*M (Ctrl), 0.1 *μ*M, and 1.0 *μ*M timcodar during the 9 days of the adipogenesis procedure. To compare the effect of timcodar with other macrolide drugs on adipogenesis we treated with 1 *μ*M FK506, 1 *μ*M rapamycin, and 1 *μ*M timcodar during the 9 days of the adipogenesis procedure. Total RNA extracted from Nile Red stained cells was used for real-time PCR analysis (see below).

### 2.4. Quantitative Real-Time PCR Analysis

Total RNA was extracted from mouse tissues using 5-Prime PerfectPure RNA Cell Kit (Fisher Scientific Company, LLC). Total RNA was read on a NanoDrop 2000 spectrophotometer (Thermo Fisher Scientific, Wilmington, DE), and cDNA was synthesized using High Capacity cDNA Reverse Transcription Kit (Applied Biosystems). PCR amplification of the cDNA was performed by quantitative real-time PCR using TrueAmp SYBR Green qPCR SuperMix (Advance Bioscience). The thermocycling protocol consisted of 10 min at 95°C, 40 cycles of 15 sec at 95°C, 30 sec at 60°C, and 20 sec at 72°C and finished with a melting curve ranging from 60 to 95°C to allow distinction of specific products. Normalization was performed in separate reactions primer sequences in Table 1.

### 2.5. Gel Electrophoresis and Western Blotting

Whole cell extracts (WCE) were prepared by freezing the cell pellet overnight at −80°C. The pellet was then resuspended in 3 volumes of WCE buffer (20 mM HEPES, 0.42 M NaCl, 0.2 M EDTA, and 25% glycerol; pH 7.4) plus protease inhibitor cocktail and incubated on ice for ten min followed by 100,000 ×g centrifugation at 4°C. Protein samples were resolved by SDS polyacrylamide gel electrophoresis and electrophoretically transferred to Immobilon-FL membranes. Membranes were blocked at room temperature for 1 hour in TBS [TBS; 10 mM Tris-HCl (pH 7.4) and 150 mM NaCl] containing 3% BSA. Subsequently, the membrane was incubated overnight at 4°C with antibodies to PPAR*γ* (Santa Cruz, 7273), C/EBP*α* (Santa Cruz, 365318), or heat-shock protein 90 (HSP90) (Santa Cruz, 13119) (Santa Cruz Biotechnology, Dallas, Texas). After three washes in TBST (TBS plus 0.1% Tween 20), the membrane was incubated with an infrared anti-rabbit (IRDye 800, green) or anti-mouse (IRDye 680, red) secondary antibody labeled with IRDye infrared dye (LI-COR Biosciences) for 2 hours at 4°C. Immunoreactivity was visualized and quantified by infrared scanning in the Odyssey system (LI-COR Biosciences).

### 2.6. Statistical Analysis

Data were analyzed with Prism 5 (GraphPad Software, San Diego, CA) using analysis of variance combined with Tukey's posttest to compare pairs of group means or unpaired *t*-tests. *p* values of 0.05 or smaller were considered statistically significant.

## 3. Results and Discussion

In this investigation, we show for the first time that timcodar can inhibit lipid accumulation in a cellular model of adipogenesis. We have previously shown that FKBP proteins, FKBP52 and FKBP51, have a differential effect on nuclear receptor-regulated gene activity [2, 3, 9, 35]. We have also demonstrated that targeting the FKBP proteins can alter lipid accumulation [1–4]. In this study, we utilized three immunophilin drugs, FK506, rapamycin, and timcodar (Figure 1). As stated above, FK506 and rapamycin are known to bind both FKBP12 and the larger TPR-containing FKBPs, such as FKBP52 and FKBP51. In contrast, timcodar was developed as a non-FKBP12-binding immunophilin [30]. The absence of binding to FKBP12 may result from structural differences amongst the immunophilin drugs. Both FK506 and rapamycin are macrolides, which are defined by the presence of a large macrocyclic lactone and a pyranose moiety. Timcodar lacks these specific requirements; however the compound possesses functionalities to mimic structural features of the above macrolides FK506 and rapamycin. Despite some similarity, timcodar does not contain a macrocyclic ring, a distinct difference that may explain why timcodar does not bind FKBP12 (Figure 1, presence and absence of “macrocyclic linkage”). The most characterized FKBP protein has been FKBP12, which in T lymphocytes is the target for immunosuppressant activity [13, 36]. Additionally, the FK506-FKBP12 and rapamycin-FKBP12 interaction also cause suppression of mTOR activity, resulting in the inhibition of protein translation. The FK506-FKBP12 complex elicits immunosuppression by inhibiting calmodulin-dependent phosphatase activity of calcineurin, a type 2B calcium/calmodulin-dependent phosphoserine/phosphothreonine protein phosphatase [12]. On the other hand, the rapamycin-FKBP12 complex is an immunosuppressant but had no effect on calmodulin-dependent phosphatase activity [12]. FK506 inhibition of calcineurin activity enhances adipocyte differentiation and increases lipid accumulation [14]. FKBP51 has been shown to inhibit calcineurin [23], which may be a primary method of how it increases lipid accumulation. On the other hand, mTOR signaling may be regulated by FKBP51, as it has been shown to compete with FKBP12 for inhibition [22]. Schreiber et al. recently showed that rapamycin binds to FKBP51 and inhibits signaling pathways such as mTOR for growth [22] and has been shown to decrease lipid accumulation in cellular models of adipogenesis [10, 17].

**Figure 2:**
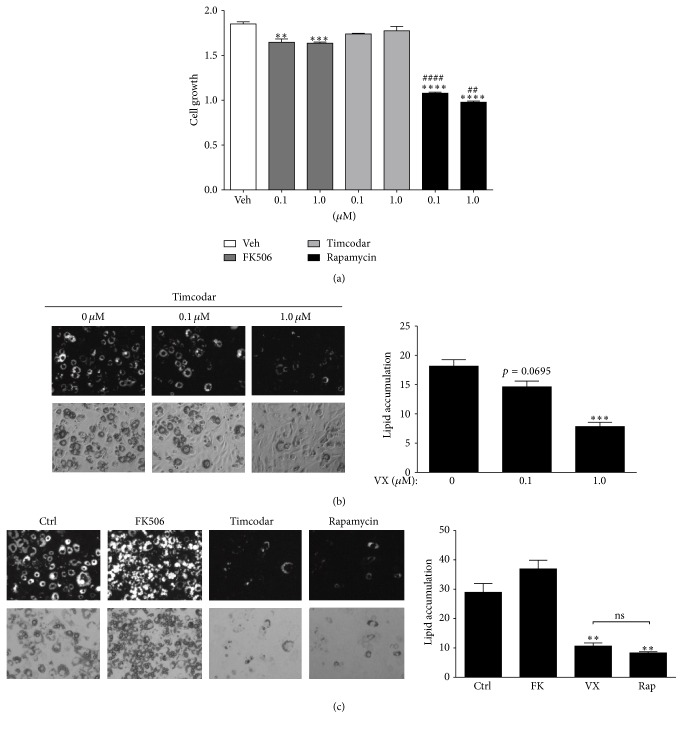
Rapamycin and timcodar reduce lipid accumulation in 3T3-L1 adipocytes. (a) Cell growth of 3T3-L1 cells in regular BCS growth serum for 48 hours with vehicle (Ctrl), 0.1 and 1.0 *μ*M FK506, 0.1 and 1.0 *μ*M timcodar, and 0.1 and 1.0 *μ*M rapamycin (ANOVA, *p* < 0.0001). Growth was measured as MTT. ^*∗∗*^ *p* < 0.01; ^*∗∗∗*^ *p* < 0.001; ^*∗∗∗∗*^ *p* < 0.0001 (versus control); ^##^ *p* < 0.01; and ^####^ *p* < 0.0001 (timcodar versus rapamycin treatment) (±S.E.; *n* = 4). (b) Nile Red staining of lipid accumulation in differentiated 3T3-L1 adipocytes treated with vehicle (0 *μ*M) and increasing doses of timcodar (VX) (0.1 *μ*M or 1.0 *μ*M); ^*∗∗∗*^ *p* < 0.001 (versus control) (±S.E.; *n* = 3). (c) Comparison of lipid accumulation of differentiated 3T3-L1 adipocytes treated with vehicle (Ctrl), 1 *μ*M FK506 (FK), 1 *μ*M timcodar (VX), and 1 *μ*M rapamycin (Rap); ^*∗∗*^ *p* < 0.01 (versus control) (±S.E.; *n* = 3).

Based on the above, we reasoned that timcodar, like rapamycin, may also regulate adipogenesis and lipid accumulation. As a first test, the effects of FK506, rapamycin, and timcodar on growth inhibitory properties and toxicity of 3T3-L1 cells were measured by treating with 0.1 and 1.0 *μ*M of each of the compounds during an MTT assay (Figure 2(a)). FK506 and rapamycin significantly suppressed growth at all concentrations. Timcodar (0.1 and 1.0 *μ*M), however, had no effect on growth inhibition. Next, to determine the impact of timcodar on lipid accumulation, we treated the 3T3-L1 adipocytes with 0.1 and 1.0 *μ*M (Figure 2(b)), which did significantly inhibit lipid accumulation at 1 *μ*M. A comparison of rapamycin (1 *μ*M) and timcodar (1 *μ*M) showed that they both significantly (*p* < 0.01) decreased lipid accumulation (Figure 2(c)). There was no significant (*p* = 0.1318) change in lipid accumulation with FK506 (1 *μ*M), even though the data were slightly elevated.

**Figure 3:**
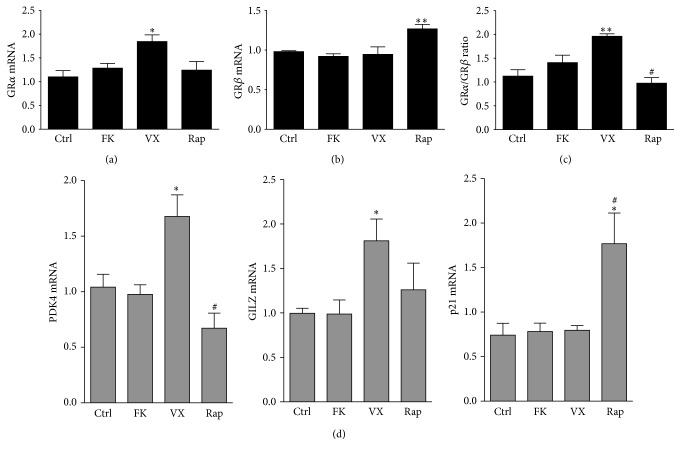
Timcodar increases glucocorticoid responsiveness by increasing the GR*α* to GR*β* ratio. Measurement of GR*α* and GR*β* mRNA was performed by real-time PCR analysis of differentiated 3T3-L1 adipocytes treated with vehicle (Ctrl), 1 *μ*M FK506 (FK), 1 *μ*M timcodar (VX), and 1 *μ*M rapamycin (Rap). (a) GR*α*, (b) GR*β*, and (c) the ratio of GR*α*/GR*β*. ^*∗*^ *p* < 0.05; ^*∗∗*^ *p* < 0.01 (versus control); ^#^ *p* < 0.05 (timcodar versus rapamycin treatment) (±S.E.; *n* = 3). (d) mRNA of glucocorticoid responsive genes PDK4, GILZ, and p21. ^*∗*^ *p* < 0.05 (versus control); ^#^ *p* < 0.05 (timcodar versus rapamycin treatment) (±S.E.; *n* = 3).

We have previously shown that timcodar's most likely target is FKBP51 because it rescued glucocorticoid (GC) signaling in FKBP52 knockout MEF cells that retained normal amounts of FKBP51 [33]. In comparison, FKBP51 knockout cells have decreased lipid accumulation [2, 3], suggesting that timcodar may have a significant impact on adipogenic signaling through FKBP51 inhibition and sensitization to GCs. The responsiveness of GCs can be determined by measuring the activity of the GC receptor (GR) at known regulated genes [37]. The GR gene (NR3C1) is complex and is only a single copy that is alternatively spliced to create multiple isoforms: GR*α*, GR*β*, GR*γ*, GR-A, and GR-P [38]. GR*α* is the classic receptor type that is known to bind to GCs and activate or suppress genes at the GC response element (GRE) in their promoters [37]. In contrast, GR*β* does not bind GCs and inhibits GR*α* [37, 39–41]. Therefore, the ratio of GR*α* to GR*β* (GR*α*/GR*β*) can assist in determining GC sensitivity [37, 39–41]. Rapamycin treatment increased GR*β* mRNA, but no change in the GR*α*/GR*β* ratio was observed (Figures 3(a)–3(c)). FK506 had no effect on GR*α* or GR*β* expression. Timcodar, however, did significantly (*p* = 0.0184) increase GR*α* mRNA, but no change in GR*β* expression, which did result in a significant increase in the ratio of GR*α*/GR*β* (*p* = 0.0043). More importantly, GC-responsive genes pyruvate dehydrogenase kinase (PDK4) and glucocorticoid-induced leucine zipper (GILZ) were significantly (*p* < 0.05) increased with timcodar treatment (Figure 3(d)), which was higher than rapamycin. FK506 had no change on PDK4, GILZ, or p21 expression. Rapamycin increased p21 higher than control, FK506, or timcodar. The antiproliferative properties of rapamycin may be attributed to elevation of the cell cycle arrest protein, p21 [42, 43]. Rapamycin is a known inhibitor of proliferation and did significantly (*p* < 0.0001) suppress growth in the MTT assay in Figure 2(a). The antiadipogenic effects of timcodar may be partially attributed to increased GILZ expression. GILZ has been shown to be an inhibitor of adipogenesis in mesenchymal stem cells, 3T3-L1 and C3H10T1/2 adipocytes [44, 45]. GILZ inhibits PPAR*γ* transcription by binding directly to the promoter, which reduces adipocyte differentiation [44].

**Figure 4:**
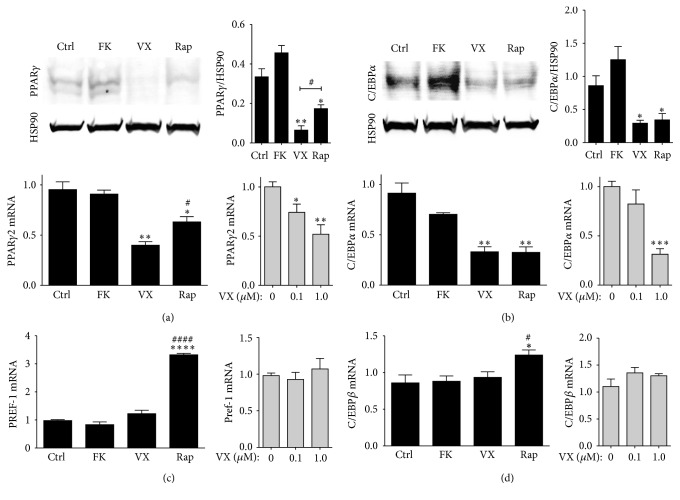
Timcodar and rapamycin reduce expression of adipogenic regulators. Western blot and densitometry for (a) PPAR*γ* and (b) C/EBP*α*, as well as real-time PCR analysis of differentiated 3T3-L1 adipocytes treated with vehicle (Ctrl), 1 *μ*M FK506 (FK), 1 *μ*M timcodar (VX), and 1 *μ*M rapamycin (Rap): (a) PPAR*γ*, (b) C/EBP*α*, (c) PREF-1, and (d) C/EBP*β*. Real-time PCR for a dose-dependent increase of timcodar for treatments of 0 *μ*M, 0.1 *μ*M, or 1.0 *μ*M in gray boxes: (a) PPAR*γ*, (b) C/EBP*α*, (c) PREF-1, and (d) C/EBP*β*. ^*∗*^ *p* < 0.05; ^*∗∗*^ *p* < 0.01; ^*∗∗∗*^ *p* < 0.001; ^*∗∗∗∗*^ *p* < 0.0001 (versus control); ^#^ *p* < 0.05; and ^####^ *p* < 0.0001 (timcodar versus rapamycin treatment) (±S.E.; *n* = 3).

The process of adipogenesis is mediated by two major regulators, PPAR*γ* and C/EBP*α*. Drugs that inhibit the PPAR*γ*-C/EBP*α* axis are considered to be antiadipogenic and reduce lipid accumulation. In Figures 4(a) and 4(b), rapamycin and timcodar inhibited PPAR*γ* and C/EBP*α* protein and mRNA expression, demonstrating that they are antiadipogenic, at least in part by suppression of the PPAR*γ*-C/EBP*α* axis. Interestingly, timcodar (1 *μ*M) robustly inhibited expression of PPAR*γ* protein and mRNA, and it was more significant than rapamycin (protein, *p* = 0.0212; mRNA, *p* = 0.0218). However, C/EBP*α* was suppressed at similar levels by rapamycin and timcodar. In contrast, FK506 (1 *μ*M) treatment did not alter PPAR*γ* and C/EBP*α* expression. Interestingly, Pref-1 was not affected by FK506 or timcodar but was induced by rapamycin (1 *μ*M) (Figure 4(c)). Pref-1 has been shown to be an inhibitor of adipogenesis [46, 47], which may be a pathway mediated by rapamycin to inhibit lipid accumulation. Cytokine production from adipocytes may cause a low-grade inflammatory state in the obese that may eventually lead to an insulin-resistant phenotype. The expression of cytokines, such as TNF*α* and IL-6, is regulated by C/EBP*β* [48, 49]. C/EBP*β* is an early adipogenic response gene that is later reduced to normal levels after an adipocyte matures [50]. Both FK506 and timcodar had no effect on expression of C/EBP*β* (Figure 4(d)). However, rapamycin (1 *μ*M) did significantly (*p* = 0.0415) enhance C/EBP*β* expression in mature adipocytes. Possibly, long-term rapamycin treatment in animals may cause insulin resistance by chronically increasing C/EBP*β*, or by GR*β* [37]. A study by Chang et al. showed that long-term rapamycin administration in mice on a high-fat diet had an adverse effect on blood glucose in rodents [51]. The insulin resistance induced by rapamycin can be reversed with resveratrol [52], which suggests that reactive oxygen species (ROS) may be involved. Antioxidants reduce obesity by preventing free radical release [53, 54], which in turn inhibit ROS production and immune cell signaling through decreased phosphorylation of NF-*κ*B [55]. Furthermore, Makki et al. demonstrated that the rapamycin-FKBP12 axis induced glucose intolerance by suppression of CD4+ T-cells and increased expression of CD8+ cells [56]. The altering of the immune system signaling has been shown to cause glucose intolerance [57].

**Figure 5:**
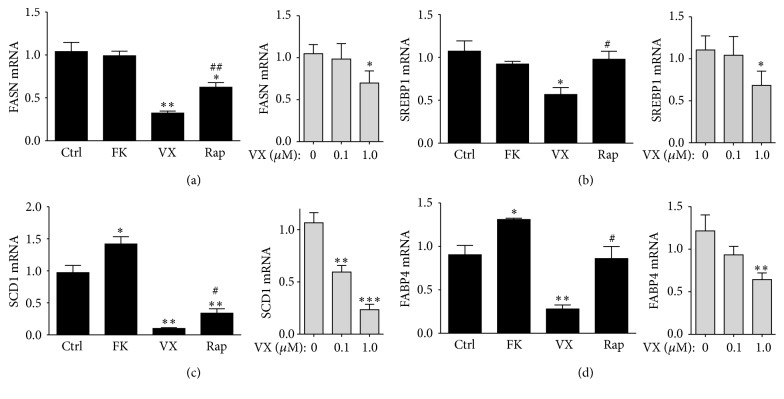
Genes involved in lipid accumulation are suppressed by timcodar. Real-time PCR analysis of differentiated 3T3-L1 adipocytes treated with vehicle (Ctrl), 1 *μ*M FK506 (FK), 1 *μ*M timcodar (VX), and 1 *μ*M rapamycin (Rap), as well as a dose-dependent increase of timcodar for treatments of 0 *μ*M, 0.1 *μ*M, or 1.0 *μ*M in gray boxes. (a) FASN, (b) SREBP1, (c) SCD1, and (d) FABP4. ^*∗*^ *p* < 0.05; ^*∗∗*^ *p* < 0.01; ^*∗∗∗*^ *p* < 0.001 (versus control); ^#^ *p* < 0.05; and ^##^ *p* < 0.01 (timcodar versus rapamycin treatment) (±S.E.; *n* = 3).

Several studies have reported that rapamycin has antiadipogenic properties that reduce lipid accumulation [52, 56, 58, 59]. Rapamycin inhibits genes that are involved in fatty acid metabolism and storage, which are essential to the adipogenic process and regulate the accumulation of lipids. Timcodar (1.0 *μ*M) treatment suppressed genes involved in* de novo* lipid production and lipid storage, such as fatty acid synthase (FASN), sterol regulatory element binding protein 1 (SREBP1), stearoyl-CoA desaturase 1 (SCD1), and fatty acid binding protein 4 (FABP4) (Figures 5(a)–5(d)). Rapamycin (1 *μ*M) treatment did suppress the expression of FASN and SCD1 but did not affect the expression of SREBP1 or FABP4. FK506 (1 *μ*M) significantly (*p* = 0.0491) increased SCD1 expression (Figure 4(c)). The impact of timcodar on lipid accumulation is most likely from the capacity of the compound to reduce the PPAR*γ*-C/EBP*α* axis. However, the effect of timcodar on obese patients or rodents is unknown.

## 4. Conclusions

Timcodar is a potent inhibitor of the adipogenic process that may prove useful in future therapies. Several studies have reported that rapamycin has antiobesity properties. However, long-term rapamycin administration may induce insulin resistance and possibly type II diabetes. Importantly, timcodar does not bind to the small FKBP proteins, such as FKBP12, which is necessary for inhibition of mTOR and the immune system. We did find that timcodar robustly inhibited lipid accumulation, by suppression of genes that regulate the adipogenic process (PPAR*γ* and C/EBP*α*), and* de novo* lipid production and storage. Inhibiting the expansion of adipocytes in the obese provides an avenue to regulate body mass size. Although its mechanism of action remains unresolved, these results suggest that timcodar may serve as a new therapeutic in the treatment of obesity, which is rapidly intensifying as an epidemic.

## Figures and Tables

**Table 1:** Primer sequences.

Primer name	Forward sequence	Reverse sequence
GR*α*	AAAGAGCTAGGAAAAGCCATTGTC	TCAGCTAACATCTCTGGGAATTCA
GR*β*	AAAGAGCTAGGAAAAGCCATTGTC	CTGTCTTTGGGCTTTTGAGATAGG
PDK4	TTTCTCGTCTCTACGCCAAG	GATACACCAGTCATCAGCTTCG
GILZ	AATGCGGCCACGGATG	GGACTTCACGTTTCAGTGGACA
p21	TGAATGGAGACAGAGACCCCA	GGAACAGGTCGGACATCACC
PPAR*γ*2	AAACTCTGGGAGATTCTCCTGTTG	GAAGTGCTCATAGGCAGTGCA
C/EBP*α*	AGAGCCGAGATAAAGCCAACA	GCAGGCGGTCATTGTCACT
PREF-1	CGGGAAATTCTGCGAAATAG	TGTGCAGGAGCATTCGTACT
C/EBP*β*	TTATAAACCTCCCGCTCGGC	TTCCATGGGTCTAAAGGCGG
FASN	GCTGCTGTTGGAAGTCAGC	AGTGTTCGTTCCTCGGAGTG
SREBP1	CACCAGCATAGGCGAAGGA	AGTGTGCGGCCTGTGGAT
SCD1	GTACCGCTGGCACATCAACT	AACTCAGAAGCCCAAAGCTCA
FABP4	GTCACAGCACCCTCCTGAAA	GGCAAAGCCCACTCCTACTT
GAPDH	CAATGTGTCCGTCGTGGATCT	GTCCTCAGTGTAGCCCAAGATG
